# Crimean-Congo Hemorrhagic Fever Virus (CCHFV): A Silent but Widespread Threat

**DOI:** 10.1007/s40475-021-00235-4

**Published:** 2021-03-16

**Authors:** Paul A. Kuehnert, Christopher P. Stefan, Catherine V. Badger, Keersten M. Ricks

**Affiliations:** 1 Diagnostic Systems Division, US Army Medical Research Institute of Infectious Diseases, 1425 Porter St, Frederick, MD 21702 USA; 2 Virology Division, US Army Medical Research Institute of Infectious Diseases, 1425 Porter St, Frederick, MD 21702 USA

**Keywords:** Crimean-congo hemorrhagic fever, *Hyalomma*, Sentinel animals, Vector spread, Biosurveillance

## Abstract

**Purpose of Review:**

This review is aimed at highlighting recent research and articles on the complicated relationship between virus, vector, and host and how biosurveillance at each level informs disease spread and risk.

**Recent Findings:**

While human cases of CCHFV and tick identification in non-endemic areas in 2019–2020 were reported to sites such as ProMed, there is a gap in recent published literature on these and broader CCHFV surveillance efforts from the late 2010s.

**Summary:**

A review of the complex aspects of CCHFV maintenance in the environment coupled with high fatality rate and lack of vaccines and therapeutics warrants the need for a One-Health approach toward detection and increased biosurveillance programs for CCHFV.

## Introduction

Crimean-Congo Hemorrhagic Fever virus (CCHFV) is a tick-borne pathogen belonging to the *Nairoviridae* family within the *Bunyavirales* order [1]. The virus was first discovered in the 1940s when Soviet soldiers became ill with a hemorrhagic disease after occupying Crimea [2]. In the 1960s, a virus with identical clinical manifestations was discovered in the Belgian Congo (now known as the Democratic Republic of Congo) and was determined to be antigenically identical to that of the virus discovered in Crimea, thus giving rise to the name CCHFV [3]. As with all Bunyaviruses, its genome is tripartite consisting of single-stranded (−) RNA segments—annotated based on length as small (S), medium (M), and large (L)—and due to each segment possessing complementary 5′ and 3′ ends, the genome forms iconic circular (panhandle) structures [4, 5]. The segments encode nucleocapsid (N protein), glycoprotein precursor (GPC), and RNA-dependent RNA polymerase, respectively [6]. CCHF virions are spherical with a diameter of 80 to 100 nm with an envelope studded with glycoproteins (GPs) G_n_ and G_c_ [7].

CCHF is the most widespread tick-borne human disease due to the extensive geographical distribution of its vector, the *Hyalomma* tick [4, 8]. Distribution of the virus matches that of hard ticks (ixodid) with the genus *Hyalomma* being the main vector. Transmission to humans occurs through tick bites, tick crushing, and contact with infected blood or tissues. Infection in human can result in mild to severe manifestations with severe cases resulting in hemorrhagic disease and a fatality rate from 5 to 30%. After a short incubation period, commonly a week, CCHFV infection is marked by common infection symptoms of high fever, malaise, myalgia, and often gastrointestinal distress [9]. Disseminated intravascular coagulopathy, shock, and/or multi-organ failure are common endpoints for a fatal outcome of the disease. In fatal human cases, CCHFV is found in many tissues including the spleen, heart, lung, and intestine. Chief cellular targets of infection are mononuclear phagocytes, endothelial cells, and hepatocytes [10]. The viral load in patients has been reported at 10^8^–10^9^ copies/mL with fatal cases purported to be slightly higher and maintained until succumbing [11]. Immune response in the form of IgM and IgG production usually develops within 7–9 days of infection in humans. IgM and IgG antibodies are detectable for months or years after infection, respectively. However, failure to mount an antibody response almost always corresponds to a fatal outcome [12].

Because of the widespread nature of the vector, high fatality rate, and the lack of medical countermeasures for treatment/prevention of disease, CCHFV is characterized as a high priority pathogen by the World Health Organization. There is growing concern of CCHFV being introduced to previously naïve areas as tick distribution extends through a combination climate change, anthropogenic factors, and transportation on infested birds, imported livestock, or both [13]. In this brief review, we discuss the literature on the enzootic vertebrate-tick-vertebrate cycle, domesticated animals as silent sentinels of CCHFV circulation in the environment, recently reported human cases and what risk factors lead to increased exposure, and how the further spread of the tick vector due to environmental changes could change the landscape of CCHFV transmission in humans.

## Vectors, Hosts, and Sentinel Animals

**Fig. 1:**
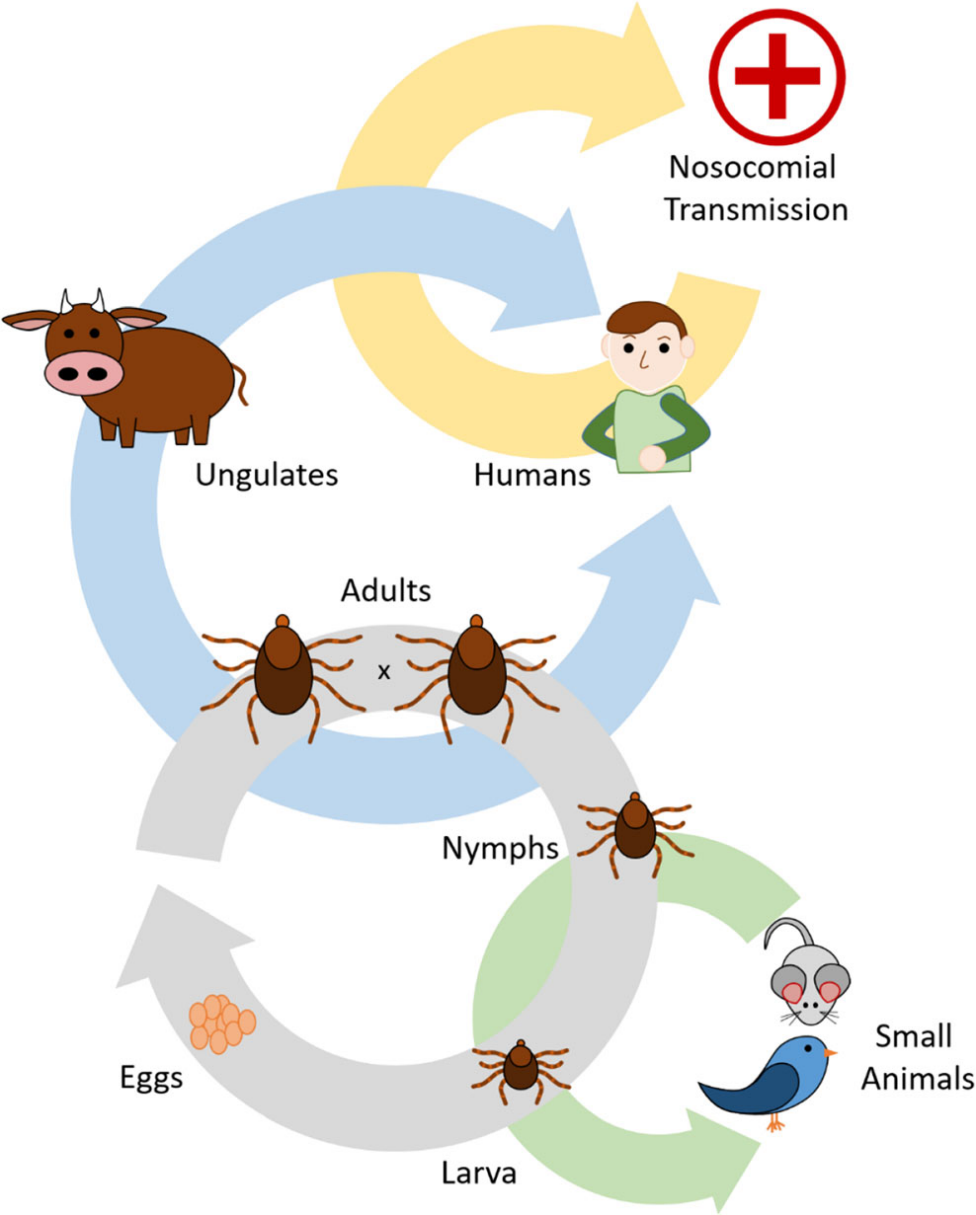
Life cycle and transmission of *Hyalomma spp* ticks. *Hyalomma spp.* maintain a two-host life-cycle, initially molting from larva to nymphs on small animals, such as birds and rodents, then transitioning to larger vertebrate, including humans. Direct contact with CCHFV infected ticks, infected non-human vertebrates, and nosocomial transmission represent significant sources of human infection. The effects of increasing human movement, vertebrate host migration, and climate change which affects migratory habits of small animals threatens to increase CCHFV infections within the population

The species of *Hyalomma*, predominantly responsible for transmission of CCHFV, is dependent on geographic region—for example, *marginatum* and *asiaticum* in Europe and Asia, respectively [14]. The vector is found throughout Africa, Southern and Eastern Europe, the Middle East, India, and Asia. Ixodid ticks, which include the *Hyalomma spp.* CCHFV vector, progress through 3 morphological stages—larva, nymph, and adult—molting at each transition and acquiring nutrients by feeding for days or even weeks on a host during each stage [15]. *Hyalomma spp.* maintain a two-host lifecycle, molting from larva to nymph on their first host—commonly a small mammal or ground dwelling bird, then to non-human and/or human vertebrates in the adult stage of the life cycle (Fig. 1) [16]. Though CCHFV has been detected in a plethora of tick species, this does not mean all are competent vectors. For a tick to contribute to the CCHFV tick-host-tick lifecycle, several conditions must be met. First, the tick must be able to acquire the virus during a blood meal. Second, the tick’s cells must support viral replication and subsequently transmit to another vertebrate host. Since ticks only feed once per developmental stage, a competent host must also maintain infection through molting (horizontal transfer). Additionally, viruses can be transferred from one generation to the next sexually or transovarial (vertical transfer). As such, CCHFV is maintained through both horizontal and vertical means.

Recent reports suggest the extended interplay between virus and tick has a significant effect on viral genome plasticity and selection, highlighting the importance of understanding this interface [17, 18]. CCHFV infection has been shown to persist throughout the tick’s lifecycle with no deleterious effect on the tick itself, further complicating the equation of CCHFV persistence in the enzootic cycle [19••]. Tick vectors can even function as a biological storage container, allowing CCHF to over-winter. *H. marginatum* was able to transmit the virus to a vertebrate host after 10 months at 4C [20]. Long-term survival in the tick vector is important for disease spread when small animal vertebrate host turn over in the ecosystem is short-lived [19••].

Animals are an essential part of the obligate, ectoparasitic lifecycle of ticks and have become fundamental to virus propagation in the tick reservoir. Vertebrates—humans are an exception—are asymptomatic during a CCHF infection but maintain a short period of viremia lasting less than 14 days [21]. This short window of infection is sufficient for viral propagation but poses significant challenges to monitoring active CCHF infections in livestock and other sentinel animal populations. Nucleic acid detection diagnostic methods—including PCR, recombinase polymerase amplification (RPA), and sequencing—have limited utility in surveillance due to the volume of samples required to find “the needle in the haystack”.

We find clues pointing to the complexity of the enzootic cycle of CCHFV in several tick collection studies. During 2013–2015, ticks were collected from 86 collection points across Turkey and identified morphologically. In total 3098 adults, 215 nymphs, and 970 larvae were collected from domestic and wild animals, humans, and ground collection. CCHFV was detected in ticks obtained from cattle, goats, wild boar, hare, and the ground, but none were found in those collected from humans, donkeys, dogs, and other small animals. The study prioritized questing ticks and allowing larvae and nymphs to molt after collection to better establish which species have horizontal transfer potential and thus possible vector competency. By surveying a diverse group of vertebrates, hints of previous unknown animals to the enzootic cycle can be discovered [22]. In an excellent 2017 review by Gargili et al., 109 studies were identified between the years of 1970 to 2016 that report the presence of CCHFV in *Hyalomma spp.* collected while feeding on hosts [19••]. CCHFV was found in other non-*Hyalomma* ticks, but the author’s stress that more research is required to fully understand viral circulation in non-*Hyalomma* ticks before inferring vector competency and further implications on public health.

## CCHFV in Humans and Occupational Risk Factors

Given the silent nature of CCHFV in the vector and non-human vertebrate hosts, the virus remains largely unnoticed until human cases arise, hallmarked by a sudden onset of symptoms [1]. While CCHFV is considered a tick-borne disease, incidence of human infection is not solely attributed to tick bites [23, 24]. In a review article written by Ergönül in 2006, a little over 3400 human cases of CCHF had been reported since 1945, with the majority of cases originating in Southeast Europe, followed by Asia, the Middle East, and Africa in numbers of cases [25]. A 2019 review from Fillâtre et al. reports over 11,000 suspected and confirmed human cases reported since 2000, where 10,000 of those cases were from the Ministry of Health in Turkey over a 15-year period [26, 27]. A search of The Program for Monitoring Emerging Diseases (ProMed) online database yielded well over 100 reported human cases of CCHFV in 2019 alone in countries such as Pakistan, Uganda, South Africa, Iran, India, Namibia, and Russia and other countries in Africa/Middle East/Asia (Table 1). Similar human case trends were reported in 2020, including cases in Spain and Bulgaria, to the surprise of the ministries of health in those countries.

**Table 1:** CCHF Human Cases and Vector Evidence from 2019 to 2020. ProMed was queried for any posts reporting on CCHF cases and/or *Hyalomma* tick identification from a period of 1/1/2019 to 12/31/2020

Country	Year	Case number (deaths)	Notes
Bulgaria	2020	1	Ministry of Health announced one case of CCHF, no other details available
India	2019	37 (19)	Majority of cases from the Gujarat region in west India, near Pakistan
	2020	4 (1)	
Iran	2019	119 (11)	
	2020	38 (5)	Represents cases from March to August
Kazakhstan	2020	1	
Mali	2020	14 (7)	
Namibia	2019	1	6 additional cases were deemed presumptive but tested negative
Oman	2019	1	Ministry of Agriculture and Fisheries imposed quarantine of farm where case originated
Pakistan	2019	51 (19)	Only cases of Karachi and Balochistan regions are represented.
	2020	7 (2)	Low numbers possibly represent a decrease in reporting on CCHF cases during COVID-19 pandemic
Russia	2019	38	Reported from the Stavropol region
	2020	1	
Senegal	2019	1	
	2020	1	
South Africa	2019	3	Northern Cape, North West, and Free State each reported one case
	2020	1	
Spain	2020	2(1)	Both cases from the Salamanca region and correspond to the 3rd and 4th cases in Spain
Turkey	2020	480(15)	Cases reported for only the first half of 2020
Uganda	2019	2(2)	
	2020	1	4 other suspected cases at the time of the report
U.A.E.	2019	1	
Country	Year	Tick Count	Notes
Netherlands	2019	3	CCHFV negative
Germany	2019	50+	CCHFV negative; evidence that ticks are overwintering in Germany
England	2019	1	First evidence of Hyalomma spp. in the UK

While the overall case fatality rate varies widely, certain occupations are linked with higher exposure risk and higher case fatality rates including agricultural occupations, health-care workers, and abattoir workers [28]. Additionally, religious holidays in countries where CCHFV is endemic, such as Eid-al-Adha, pose an increased risk for human exposure as cattle and sheep, known vertebrate hosts of CCHFV, are sacrificed [29]. No matter the region where risk is assessed, higher risk of CCHFV infection is widely associated with tick exposure (tick bite or handling tick with bare hands) or animal exposure (herders, agricultural works, abattoirs, veterinarians) [23, 24]. Nosocomial infections, where there is human-to-human transmission, can occur in a healthcare setting. In a 2019 review, 158 cases of nosocomial infections were reported in 20 countries from 1958 to 2016. Nearly all cases were symptomatic, and there was a case fatality rate of 32.4% [30]. CCHF due to travel was investigated as a lesser known risk, where only 21 cases were reported from 1960 to 2016; however, this does not negate situational awareness when traveling to and from endemic areas [31]. There is a dire need for better public health education about the risk of tick-borne diseases in these occupations as well as increased preventative measure to reduce incidence of exposure [32].

## Biosurveillance

Although an increase over the past two decades in the frequency of reported CCHF cases in vectors and vertebrates, especially in Eurasia, can likely be attributed to increased viral persistence, wider vector prevalence, and regional spikes, there is also an increased awareness and One-Health focus on detection and biosurveillance to identify areas of vector spread, non-human vertebrate host seroprevalence, and human case reporting [19, 33•]. As aforementioned, there were hundreds of suspected and confirmed human cases and several reports of *Hyalomma* tick identification in non-endemic regions reported to ProMed but not yet published in the literature highlighting the need for timely reporting to reduce community spread and identify the source of transmission. A review by Sorvillo et al. describes how crucial it is that surveillance systems be coordinated across multiple sectors and take into account a One-Health approach as data on CCHFV in humans, animals, and ticks are needed to better understand and prepare for disease spread [33•].

To date researchers have leaned heavily on serological surveillance [34]. Serologic assays that measure levels of anti-CCHFV IgM and/or IgG in an animal or human are useful as companion diagnostics for patient diagnosis by PCR (IgM detection) and surveillance of animals and humans for recent or prior exposure to the pathogen (IgM and IgG). This data provides a picture of the graphical range of CCHF in amplifying hosts and can be combined with mapping of abiotic factors and vectors to produce risk projections to direct informed health policy globally [8, 35, 36]. However, an abundance of caution is warranted when interpreting a given study and across data sets. Given animal surveillance for humoral response to CCHFV has been undertaken for over half a century, it is not surprising that antibody detection methods have evolved—making comparisons of positivity rates across long time domains dubious [37]. Fortunately, many recent studies have utilized a similar combination of in-house ELISA, species-modified commercial ELISA, and direct IFA to classify IgG positive samples [38–40]. Standardization in immunological testing methodologies should be established and encouraged for CCHF surveillance at a level that is scientifically stringent and achievable across potentially resource-limited domains reflecting the global distribution of the virus.

Another challenge for serological interpretation is the longevity of the humoral response to CCHFV infection. Although humans have immune responses lasting decades, little is known about the length of antibody response in other vertebrates [41]. Studies involving murine models of CCHF have demonstrated an adaptive immune response on the order of months [42]. Longitudinal studies using animal models in a laboratory setting are often cost prohibitive and understanding CCHF pathology during early infection remains priority [43]. In addition to the unknown canonical immune response, animals can be exposed to infected ticks repeatedly from a seasonal and lifetime perspective—these factors create analog antibody response in a population versus an easily interpreted binary one. Animals can often be utilized as sentinels of public health and biothreat agents, as has been seen with anthrax and Rift Valley Fever virus in cattle and sheep; however, CCHFV is symptomatically silent in non-human vertebrate hosts [44]. This creates a unique challenge for observing livestock health when it comes to CCHFV infection.

Well-characterized diagnostic tools are needed to screen for the virus itself as well as evidence of exposure to CCFHV through serologic testing [45]. Nucleic acid detection methods, such as PCR, are used to detect CCHFV genetic material in a tick, animal, or human. The continuous evolution of the next-generation sequencing technologies promises to deliver true agnostic sample analysis identifying all unknown pathogens [46]. Often these tests can be developed to quantitate the viral load present in a patient to assist with disease prognosis and link to patient outcome. While there are not many commercial diagnostics on the market for CCHFV, many public and private sector institutions globally have developed their own assays for CCHFV detection. These assays have been utilized for biosurveillance of animals and humans to assess regional CCHFV prevalence, as is outlined in many recent reviews [28, 34]. It is critical to form a biosurveillance strategy based on a diagnostic toolbox that utilizes both molecular and serologic detection methods in order to understand true CCHFV prevalence and human disease risk.

## Spread of CCHFV to Non-Endemic Areas

Spread of CCHFV from endemic areas has been of growing concern for many reasons, especially considering it is already the most widespread tick-borne disease. This spread can be attributed to human movement, vertebrate host migration, and climate change [47••]. Spain recorded the country’s first autochthonous case of CCHF in 2017—the case was fatal, and there was nosocomial transmission to a nurse. While the risk for CCHFV in Spain remains low based on tick and human surveillance, this etiological agent now circulating at levels capable of infecting humans indicates either a resurgence from an undetectable level or recent importation to the Iberian Peninsula [48]. Phylogenetic analysis showed clustering with Africa III clade of CCHFV, supporting the later hypothesis [49]. In 2015, an Oregon resident returned from a trip to Ethiopia with a *Hyalomma* tick attached to his lower back [50]. The tick was not infected with CCHFV; however, this report highlighted how tick species can be so easily introduced to non-endemic areas due to human movement. Six cases of human travel related importation of CCHF to non-endemic countries have been reported since the 1990s.

Ticks, especially *Hyalomma*, commonly have questing behavior to seek out hosts, but these movements are dwarfed by dispersion once attached to much larger and mobile hosts—birds, humans, and commercial undulates serve as far-reaching vehicles for ticks [51]. Birds have garnered attention recently as CHHFV-positive ticks have been collected on species in Greece, Morocco, and most recently Italy [52–54]. Although the tick burden appears low—for example, over two years (2013/2014), 50,325 birds were screened in Italy with 0.28% having tick infestations or 0.22% infested with *Hyalomma* specifically—it is estimated that 2.1 billion birds migrate from CCHF endemic areas in Africa to Europe yearly resulting in a possible importation of millions of *Hyalomma* ticks annually [16, 55]. Whether avian species can function as an amplifying host in addition to vector ride-shares is unclear—only ostriches have been successfully infected with CCHFV [56]. Movement of livestock is of concern, as there are no clinical signs of CCHFV infection in non-human vertebrates that would trigger cause for screening at a control point. Expansion of CCHFV endemicity is more of a concern in the livestock trade for countries bordering CCHFV-endemic regions, as there is not much trans-oceanic transport of these animals [47••]. All these scenarios add avenues of tick migration to non-endemic areas that should warrant heightened biosurveillance for CCHFV in addition to other tick-borne diseases.

Expansion of *Hyalomma* ticks has also been modeled under the lens of climate change [36]. It can be extremely difficult to decouple the effects of climate change on the spread of ticks and tick-borne diseases from other factors that affect vector and disease spread and/or perception of spread like human migration, urbanization, and increase in detection/biosurveillance [57]. Nonetheless, there is still interest in the last decade on how climate change affects vector and host behavior and movement, changing abiotic conditions that promote tick health and development, ecology, and vegetation changes, among many other factors [58].

## Conclusion

Given the complicated enzootic cycle and transmission dynamics of CCHFV coupled with its silence in nature until the virus has reached the human host, a One-Health approach toward biosurveillance of ticks, animals, and humans is necessary to understand maintenance in the environment and identify potential outbreaks in humans. In addition to surveillance, there needs to be a concerted effort to better public health campaigns on occupational risks associated with not only CCHFV infection but also other infectious diseases. Research institutions and ministries of health should strive to share information as quickly as possible to sites such as ProMed and other scientific outlets, so there is an increased awareness in real-time of CCHFV outbreaks. Lastly, there need to be validated diagnostic tools for use in the field and in centralized laboratories to facilitate rapid and accurate detection and diagnosis as well as periodic biosurveillance campaigns utilizing these diagnostic tools to try and stay ahead of disease spread.
